# The metatranscriptomes of root caries and sound root surface biofilms

**DOI:** 10.1080/20002297.2017.1325195

**Published:** 2017-06-09

**Authors:** Thuy Do, Naile Dame-Teixeira, Clarissa C.F. Parolo, Marisa Maltz, Aradhna Tugnait, Deirdre Devine

**Affiliations:** ^a^ School of Dentistry, University of Leeds, Leeds, UK; ^b^ Department of Dentistry – Cariology, University of Brasilia, Brasilia, Brazil; ^c^ Faculty of Dentistry, Federal University of Rio Grande do Sul, Porto Alegre, Brazil

## Abstract

There is limited knowledge of bacterial metabolism in root caries lesions. The aim of this study was to describe the bacterial metatranscriptomes associated with root caries and sound root surfaces using an RNA-seq analysis approach.

The biofilms from exposed root surfaces were sampled from caries-free volunteers (n=10), and from the infected dentine of volunteers with root caries (n=30). Total bacterial RNA was extracted; cDNA libraries were prepared and sequenced on the Illumina Hi-Seq2500. The function and composition of the metabolically active microbiota were investigated using: a) MG-RAST, and b) denovo assembly of the read data and mapping to contigs. Differential gene expression analysis was done using the R package DESeq2 (padj <10^−3^).

Transcripts with the highest expression levels were those coding for membrane transport systems, ribosomal proteins, enolase and glycolytic pathways in both groups. Differential analysis indicated that genes coding for the OmpA domain protein and metalloprotease domain protein were over-expressed in the caries samples (log_2_FoldChange = –12.2; padj= 3.5 × 10^−13^), whereas genes in the samples from healthy sites over-expressed pilus biosynthesis protein, thiamine diphosphokinase and transporter protein (log_2_FoldChange = 16.5; padj = 2.2 × 10^−21^).

Metatranscriptomic analyses show unique gene expression profiles in sound root surface and carious biofilms.

